# Controlled trial to compare the Achilles tendon load during running in flatfeet participants using a customized arch support orthoses vs an orthotic heel lift

**DOI:** 10.1186/s12891-019-2898-0

**Published:** 2019-11-13

**Authors:** Kawin K. W. Lee, Samuel K. K. Ling, Patrick S. H. Yung

**Affiliations:** 1 Department of Prosthetics and Orthotics, Queen Mary Hospital, Hong Kong, Hong Kong; 2 Department of Orthopaedics and Traumatology, Faculty of Medicine, CUHK, Hong Kong, Hong Kong

**Keywords:** Achilles tendon, Tendinopathy, Flatfeet, Runners, Orthoses, Arch support, Heel lift, Insole, Load

## Abstract

**Background:**

Achilles tendinopathy is one of the most common overuse injuries in running, and forefoot pronation, seen in flatfeet participants, has been proposed to cause additional loading across the Achilles tendon. Foot orthoses are one of the common and effective conservative treatment prescribed for Achilles tendinopathy, it works by correcting the biomechanical malalignment and reducing tendon load. Previous studies have shown reduction of Achilles Tendon load (ATL) during running by using customized arch support orthosis (CASO) or an orthotic heel lift (HL). However, there are still little biomechanical evidence and comparative studies to guide orthotic prescriptions for Achilles tendinopathy management. Therefore, this study seeks to investigate the two currently employed orthotic treatment options for Achilles tendinopathy: CASO and HL for the reduction of ATL and Achilles tendon loading rate (ATLR) in recreational runners with flatfeet.

**Methods:**

Twelve participants were recruited and run along the runway in the laboratory for three conditions: (1) without orthoses, (2) with CASO (3) with HL. Kinematic and kinetic data were recorded by 3D motion capturing system and force platform. Ankle joint moments and ATL were computed and compared within the three conditions.

**Results:**

Participants who ran with CASO (*p* = 0.001, d = 0.43) or HL (p = 0.001, d = 0.48) associated with a significant reduction in ATL when compared to without orthotics while there was no significant difference between the two types of orthoses, the mean peak ATL of CASO was slightly lower than HL. Regarding the ATLR, both orthoses, CASO (*p* = 0.003, d = 0.93) and HL (*p* = 0.004, d = 0.78), exhibited significant lower value than the control but similarly, no significant difference was noted between them in which the use of CASO yielded a slightly lower loading rate than that of HL.

**Conclusions:**

Both CASO and HL were able to cause a significant reduction in peak ATL and ATLR comparing to without orthotics condition. There were subtle but no statistically significant differences in the biomechanical effects between the two types of orthoses. The findings help to quantify the effect of CASO and HL on load reduction of Achilles tendon and suggests that foot orthoses may serve to prevent the incidence of Achilles tendon pathologies.

**Trial registration:**

NCT04003870 on clinicaltrials.gov 1 July 2019.

## Introduction

### Background

Running is a popular exercise with positive effect to physical and psychological health benefits [1]. Overuse running injuries have become more prevalent, especially for recreational runners [2]. Achilles tendinopathy is one of the most common overuse running injury, it accounts for 8–15% of all injuries in recreational runners [3, 4]. Achilles tendinopathy is a condition marked by heel pain and posterior leg stiffness along with pathological changes within Achilles tendon substance. These symptoms have been found to hinder physical function and subsequently impair athletic performance [5].

Regarding the reasons predisposing to the development of Achilles tendinopathy, different intrinsic and extrinsic risk factors have been hypothesized, yet the exact etiology of is still unclear [6]. Overuse, altered tissue vascularity, biomechanical imbalance, bacteria and genetic factors have all been linked to Achilles tendinopathy [7, 8]. Excessive abnormal Achilles tendon loading is considered as the key element to an overuse injury. The Achilles tendon is highly vulnerable to overuse injuries due to the repetitive overload it is subjected to during walking or running. Particularly in running, Achilles tendon experiences a force approximately 6–8 times of body weight, close to the maximum load tolerable by the tendon [9, 45].

Of which, altered foot biomechanics, especially excessive foot pronation, have been shown by numerus studies to increase the risk of additional loading to Achilles tendon through two mechanisms. Firstly, excessive foot pronation generates greater hindfoot eversion motion. When the hindfoot goes from a varus position at heel strike, to a valgus position in midstance, and then back during the running gait cycle, a “whipping” action on the Achilles tendon is created resulting in increased tensile forces over the medial aspect of The Achilles tendon. Repetitive whipping action may result in mircrotears in the tendon, initiating an inflammatory response [10, 11]. Ryan et al. have found that participants with Achilles tendinopathy showed greater hindfoot valgus during barefoot running in midstance than controls [12]. Secondly, excessive pronation contributes to asynchronous movement between ankle and foot segment during stance phase, this wrings out vessels in the tendon and peritendon causing vascular impairment as well as degenerative changes to the Achilles tendon. These imply that runners with a planus foot would have a higher chance of developing Achilles tendinopathy [10].

**Fig. 1:**
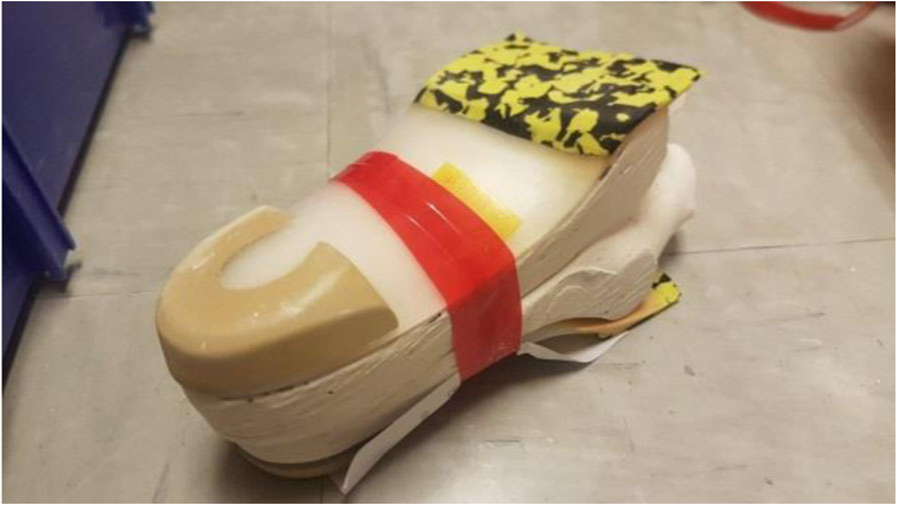
The CASO fabricated from the negative plaster cast

Among various conservative treatments, foot orthoses have long been considered as an effective intervention to conservative treatment and prevention of Achilles tendinopathy in clinical settings, with a success rates as high as 75% in runners reported from case studies and retrospective surveys [13–15]. In general, there are two commonly used foot which are the foot orthotic with medial arch support (Fig. 1) and orthotic heel lift (Fig. 4).

Although previous studies indicated that foot orthoses are an effective treatment towards Achilles tendon pathologies, there is currently insufficient evidence to explain the mechanism by which foot orthoses exert their effects when used to treat Achilles tendinopathy. The hypothesized effect of arch support orthotics in decreasing the Achilles tendon stress is the correction of biomechanical malalignment. It serves to reduce hindfoot valgus and align the calcaneus in the vertical position, thus relieving the shear stress over the Achilles tendon, particularly in the pronated foot [12, 14, 15, 17]. Besides, orthoses elevating heel height may cause plantar flexion of the ankle joint which shortens the muscle-tendon unit, thus relieving the force over Achilles tendon during gait. More recently, different biomechanical hypotheses have been proposed, including the increased of hindfoot movement variability by orthoses resulting in decreased ATL [14] and alteration of the neuromotor activity of Triceps surae towards participants with Achilles tendinopathy [18].

Load management plays a crucial role in preventing overuse Achilles tendinopathy. Previous studies [19–22] showed that both foot orthotic with medial arch support and orthotic heel lift have the ability to alter the ATL in running. However, there are still limitations of previous studies that warrant further consideration. First, there is no consensus or guidelines on the types of foot orthoses to be prescribed for Achilles tendinopathy management. Second, the foot types of participants have not been standardized, as the degree of pronation is one of the risk factors affecting the ATL, so orthotic customization for variable foot types is required to accommodate the different foot pronation among participants. Third, there are presently no comparative studies evaluating foot orthotics as the management options for Achilles tendinopathy. Moreover, there is still little biomechanical evidences regarding the alteration in ATL through orthotic intervention and comparative studies to guide orthotic prescriptions for Achilles tendinopathy management.

### Objectives

The aim of this study was to compare two clinically applied treatment options: Customized arch support orthoses (CASO) and orthotic heel lift (HL) on the effect of ATL in recreational runners with pronated feet. It aimed to provide a better understanding of the types of foot orthoses for flatfeet runners and additional biomechanical evidence for the clinical field to guide orthotic prescription as well as selection for Achilles tendinopathy management.

## Methods

### Trial design and hypothesis

This was a controlled laboratorial, within subject, repeated measures study. It was hypothesized that both orthoses would lower the ATL when compared with the no-orthotic intervention. It was also hypothesized that CASO would have a larger ATL reduction than that of HL for flatfeet runners.

### Participants

Twelve recreational runners of age greater than 18 years who trained regularly for running at least once per week and with running experience of 1 year or more [23], with excessive foot pronation and with hind-foot strike landing pattern were recruited.

Foot Posture Index (FPI) was used as evaluating pronated foot posture. FPI is a non-invasive method of assessing the degree of standing foot posture with the scores reflecting highly supinated (− 5 to − 12), supinated (− 1 to − 4), neutral (0 to + 5), pronated (+ 6 to + 9) or highly pronated (+ 10 to + 12). This is a validated instrument, which is adequately reliable as a screening tool for standing foot posture [25, 26]. Participants with FPI scores of 6–12 were recruited in the current study.

All participants should be free of Achilles tendinopathy and triceps surae injury for 6 months with no previous surgery as well as not previously attempting any foot orthoses intervention before this study. All participants were clinically assessed by the same Prosthetist and Orthotist for the range of motion, strength and flexibility of the lower extremities. Participants exhibiting leg-length discrepancy, rigid forefoot varus deformity, gastrocnemius equinus, structural hallux limitus, or rigidus were excluded. Any musculoskeletal or neurological disorders, which would affect normal running gait, were also excluded.

### Sample size

With reference to previous similar studies [14, 19, 24] and the suggestions by Chow et al. [27] to determine the sample size. Given a minimal statistical power ≥ 0.8 and α = 0.05, we are interested in an effect size of 1.2 with a standard deviation of 1. In such regard, 12 participants were considered sufficient for our study.

### Foot orthoses

#### Customized arch support orthoses (CASO)

**Fig. 2:**
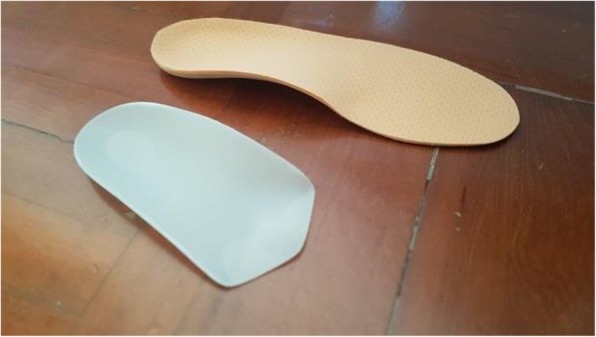
The CASO (left) and the CASO with 3 mm multiform added (right)

**Fig. 3:**
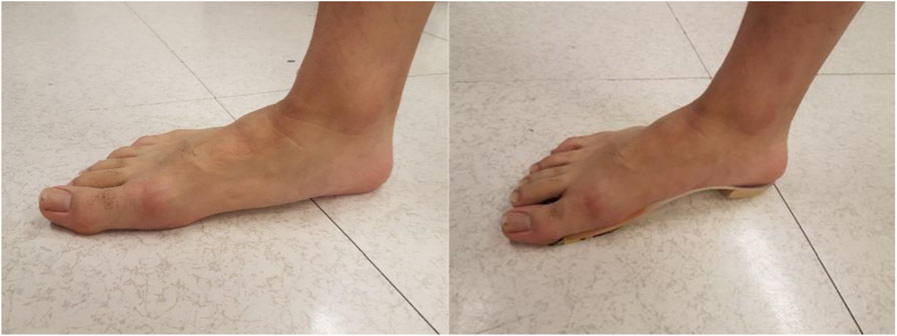
CASO intervention to foot pronation (left in barefoot condtion, right in CASO)

The CASO (Figs. 1, 2 and 3) was made using the traditional hand-made method, all participants were asked to lie prone with both feet as a non-weight bearing position and the foot plantar surface was casted by using the Plaster of Paris bandages bilaterally in a subtalar neutral position manipulated by the same Prosthetist and Orthotist. The negative casts were placed in a calcaneal vertical position and a positive mold was created from the negative cast by filling Plaster. Custom-molded orthoses were then fabricated from the positive mold with 3-mm polypropylene (Polystone® P copolymer, from Röchling) using a vacuum press method with an extrinsic ethyl vinyl acetate (EVA) standard rearfoot posting, cut at 50% of the length of the heel cup. A 3 mm multiform cover was added according to the shape of shoes.

#### Orthotic heel lift (HL)

**Fig. 4:**
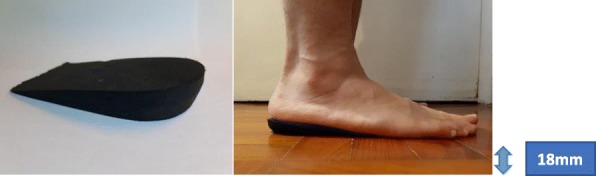
The HL with maximum height of 18 mm

The HL was made by high-density ethyl vinyl acetate (EVA), it was wedge-shaped which tapered over its 8.2 cm length from a height of 18 mm posteriorly to finish flush anteriorly. It was added underneath to the insole of the rearfoot of each shoe by double-sided adhesive tape in order to prevent any sliding movement during running (Fig. 4).

### Data collection

#### Kinematic data

**Fig. 5:**
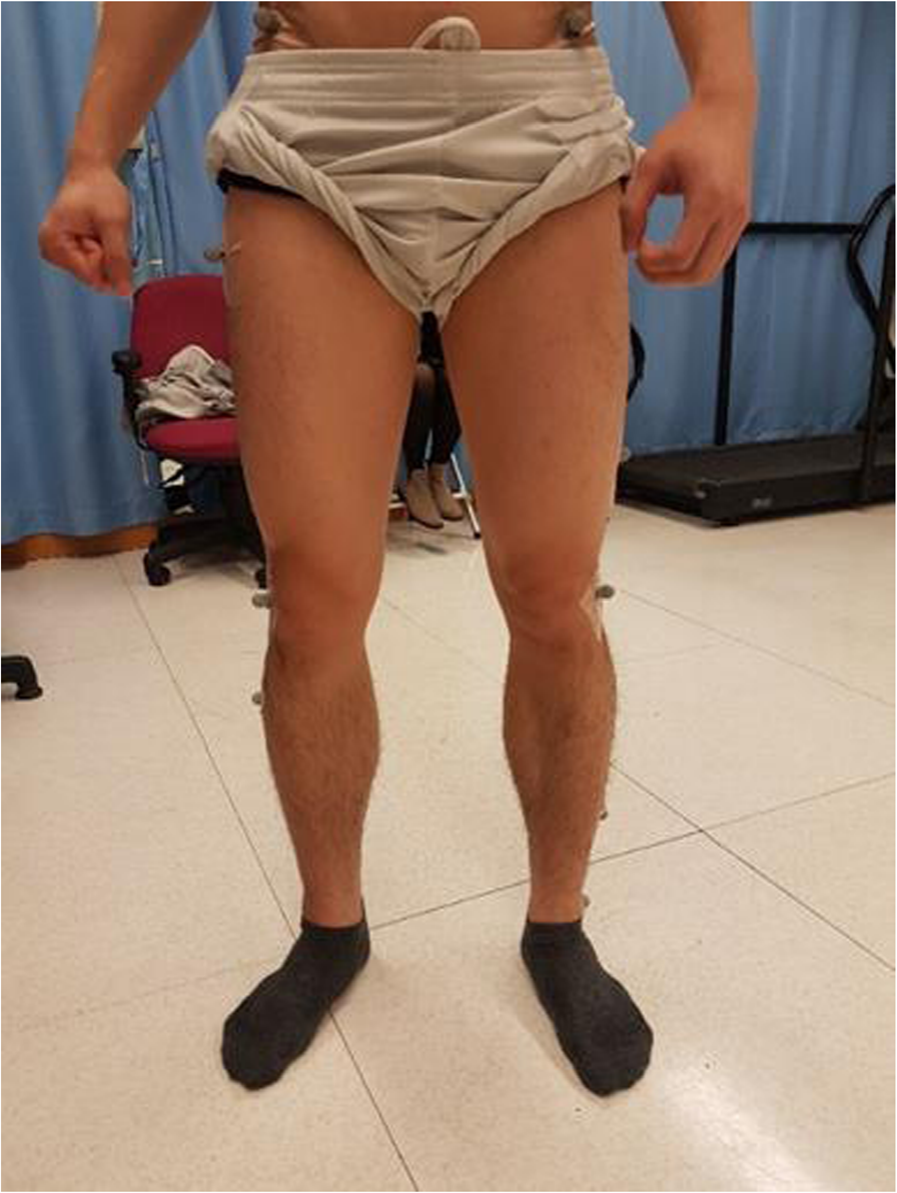
Retroreflective markers including anterior superior iliac spine, lateral thigh, lateral femoral epicondyle, lateral shank, lateral malleolus

Kinematic data was captured by an 8-camera motion capturing system (Vicon 370, Oxford, UK). Kinematic analysis and a calculation of the position of the center of mass were performed using the lower body Plug-in-Gait model. Sixteen retroreflective markers were positioned onto the posterior superior iliac spine, anterior superior iliac spine, lateral thigh, lateral femoral epicondyle, lateral shank, lateral malleolus, calcaneus and second metatarsal head (on shoes) bilaterally following anatomical landmark for defining anatomical frames of the right foot and shank (Fig. 5). The foot segment was tracked using the metatarsal and calcaneus markers. Before data collection, static calibration trials were obtained with participants in the anatomical position ensuring the anatomical markers are in reference with the technical marker positions as shown in Fig. 5.

#### Kinetic data

Piezoelectric force platform (AMTI force plates) was used for capturing kinetic data once participants strike with their right (dominant) foot. The stance phase of the running cycle was identified at the time over which ≥20 N of vertical force was applied to the force platform [28].

#### Achilles tendon loading (ATL)

Ankle joint kinetics were computed using the Newton–Euler inverse-dynamics. Net external ankle joint moments were then calculated which has been shown to give a relevant approximation for internal joint loading [29].

An algorithmic model was used as a predictive technique to determine ATL. This technique has been shown to be sufficiently sensitive to resolve differences in ATL during running with different footwear [30, 31].

Achilles tendon load (ATL) is determined by dividing the plantarflexion moment (MPF) by the estimated Achilles tendon moment arm (MA):

$$ \mathrm{ATL}=\mathrm{MPF}/\mathrm{MA} $$

The moment arm was quantified as a function of the ankle sagittal plane angle (SAK) using the procedure described by Self and Paine [32], the equation is calculated by Rugg et al. using magnetic resonance imaging (MRI) [33].

$$ \mathrm{MA}=-0.5910+0.08297\ \mathrm{SAK}-0.0002606\ {\mathrm{SAK}}^2 $$

ATL was normalized to body weight (B.W.) and Achilles tendon loading rate (ATLR) (B.W. sˉ^1^) was also calculated as a function of the change in ATL from initial contact to peak ATL divided by the time to peak ATL.

### Study settings

**Fig. 6:**
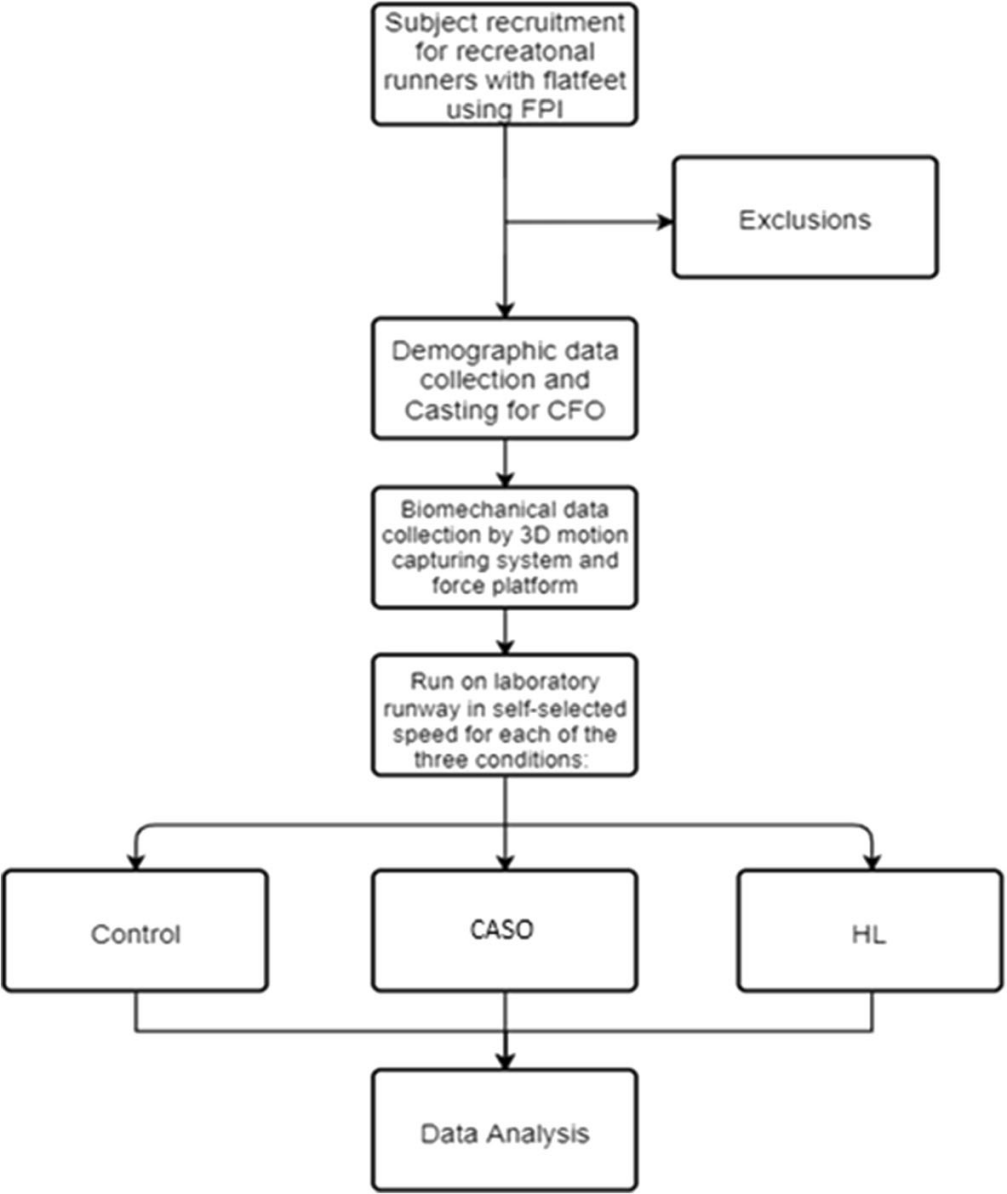
The flow of the experimental procedure

There were in total two face-to-face appointments for each subject. In the first appointment, initial screening and assessment based on the inclusion and exclusion criteria by Prosthetists and Orthotists were done. For the assessment for flatfeet by FPI, participants were required to stand in a relaxed position; the rearfoot was first assessed through palpation of the head of the talus, observation of the curves above and below the lateral malleoli and the degree of the inversion/eversion of the calcaneus. Scores would be summed according to the six parameters. Participants with FPI scores of 6–12 were recruited Then for the forefoot, the bulge in the region of the talonavicular joint, the extent of abduction/adduction of the forefoot on the rearfoot and the congruence of the medial longitudinal arch. Participants fulfilling research requirement cast for the CASO by the method mentioned above and demographic data were collected. Data collection was done in the second appointment. The second appointment was arranged once the CASO and HL were ready (which was ~ 2–3 weeks) (Fig. 6).

**Fig. 7:**
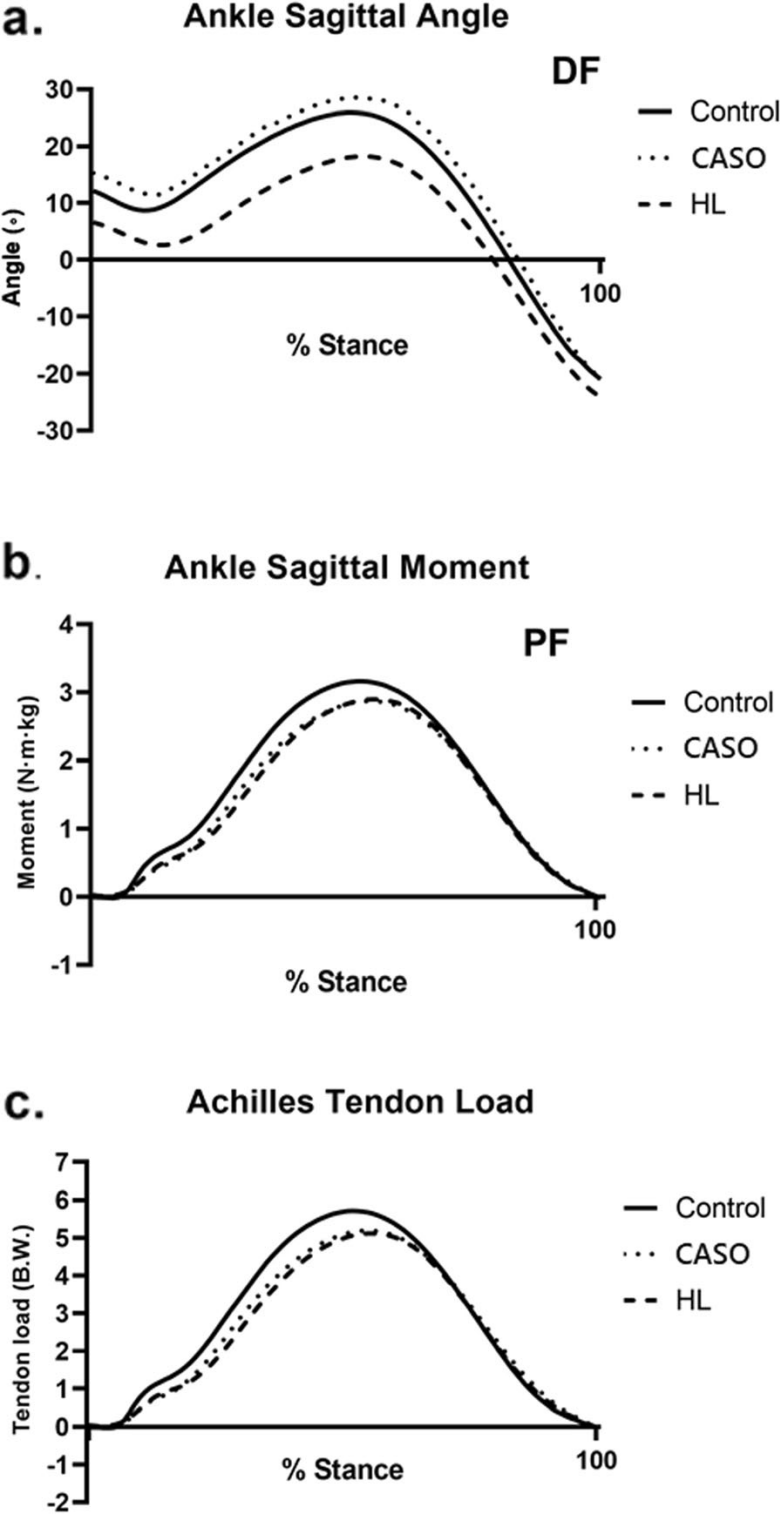
Ankle kinetics and kinematics in the three conditions during stance phase of running (a = sagittal ankle angle, b = sagittal plantarflexion moment, c = Achilles tendon load)

The experimental procedure was conducted in a 10 m long gait lab. Before testing commenced, there was a 15 min acclimatization period for each subject to walk or run on the runway. During this period, the subject walked or ran with orthoses at their comfortable speed and gait pattern to adjust to the surroundings and to make sure the orthoses were comfortable and the running gait was consistent. After this period, participants were asked if they needed more time. If they asked for more time, additional familiarization was given until they feel accustomed to the condition. There were 3 min rest before testing to avoid muscle fatigue.

### Interventions

For the testing condition, participants were asked to run on the runway at a self-selected speed in threes conditions: (1) run without orthoses, (2) run with HL and (3) run with CASO (Fig. 7). The test sequence was randomized using an online program (www.random.org) which is a computerised random number generator. Recording began once participants ran with their comfortable running pace. The eight-camera system and the force platform were used to record the kinematic and kinetic data synchronously at 250 and 1000 Hz respectively. Participants were required to complete five acceptable trials (completely contacted the force plate with the right leg with acceptable speed and without targeting) running at a self-selected speed at each condition. There were 3 min rest between each condition to minimize the carry-over effects. To minimize the effect of speed on biomechanical parameters, all actual trials were required to be within ±5% of the determined average self-selected speed [34], while the running speed was monitored by mobile phone positioned 1.5 m from the force plates using a video radar application (SpeedClock, Sten Kaiser, version 3.1) [35].

### Data processing

VICON Nexus v2.6, Oxford Metrics software (Plug-in-Gait model) was used to compute joint kinematics and kinetics. Data were exported to MatLab software (R2019a) for further processing. Kinematics and kinetics data were time normalized (0–100%) of the stance phase and averaged across five trials to get individual mean curves. Joint angles and internal joint moments (N.m.kgˉ^1^) during the stance phase of running were determined across five successful force plate contacts of the right leg. Marker trajectories and kinetic data were low-pass filtered using a fourth-order Butterworth filter with cutoff frequencies of 12 and 50 Hz respectively. Kinetic variables were all normalized for body mass.

### Statistical analysis

Normality distribution analysis was carried out within conditions for the data of the results by the Shapiro Wilks test, considering a normal distribution when *P* > 0.05. Demographic characteristics such as age, height and weight were included. Mean and standard deviation (SD) were applied to the data set to describe quantitative data. Mauchly’s test was used to assess sphericity of data. One-way repeated measures ANOVA was conducted in order to examine the differences in primary outcomes: peak Achilles tendon load (B.W.) and Achilles tendon loading rate (B.W. sˉ^1^), as well as secondary outcomes include peak plantarflexion moment, time to peak Achilles tendon force and peak dorsiflexion angle among the three conditions. Post-hoc comparisons with Bonferroni correction was used as a follow-up analysis.

All statistical tests were conducted by means of the IBM SPSS Statistics software (SPSS, v22, Inc., Chicago, Illinois). Statistically significant differences were considered at *P* < 0.05 with a 95% confidence interval (CI). Effect sizes in terms of Cohen’s d were calculated for the difference in the two groups’ means divided by the average of their standard deviations in order to quantify the differences between the three conditions (https://www.socscistatistics.com/effectsize/). The effect is regarded as small, medium and large when Cohen’s d is 0.2, 0.5 and 0.8 respectively [36].

## Results

In total, 12 participants (10 males and 2 females) were recruited (Table 1). The ankle kinetics and kinematics with respect to the three testing conditions were presented in Table 2. Pairwise comparisons for mean difference and 95% Confidence interval for difference of three conditions are listed in Table 3.

**Table 1:** Demographics of the participants (*n* = 12)

Characteristics	Mean ± Standard Deviation
Age (year)	25.3 ± 1.2
Weight (kg)	62.3 ± 13.5
Height (m)	1.69 ± 5.7
Foot Posture Index (FPI)	+ 9 ± 1.5
Running experience (year)	6.5 ± 2.5
Running distance (km/week)	11.8 ± 3.4

**Table 2:** Achilles tendon kinetics and kinematics as a function of orthotic interventions

	No orthotic		CASO		HL		*p*-value
	Mean	SD	Mean	SD	Mean	SD	
Peak plantarflexion moment (N·m·kg)	3.21	0.72	2.91	0.64	2.94	0.68	< 0.001*
Peak Achilles tendon load (B.W.)	5.80	1.29	5.27	1.18	5.20	1.21	< 0.001*
Time to peak Achilles tendon force (s)	0.15	0.11	0.17	0.14	0.16	0.17	0.001*
Achilles tendon loading rate(B.W.·sˉ^1^)	38.25	7.81	31.19	7.27	31.89	8.55	< 0.001*
Peak dorsiflexion angle (∘)	25.58	10.96	27.12	10.80	18.39	9.17	< 0.001*

Notes: * = significant difference *P* < 0.05, *B.W.* Body weight

**Table 3:** Pairwise comparisons for mean difference and 95% Confidence interval for difference

Conditions		Mean Difference	95% Confidence Interval for Difference	
			Lower Bound	Upper Bound
No orthotic	CFO	0.53	0.25	0.81
	HL	0.59	0.34	0.85
CFO	No orthotic	−0.53	−0.81	− 0.25
	HL	0.62	−0.17	0.30
HL	No orthotic	−0.59	−0.85	− 0.34
	CFO	−0.62	−0.30	0.17

### Peak plantarflexion moment

**Fig. 8:**
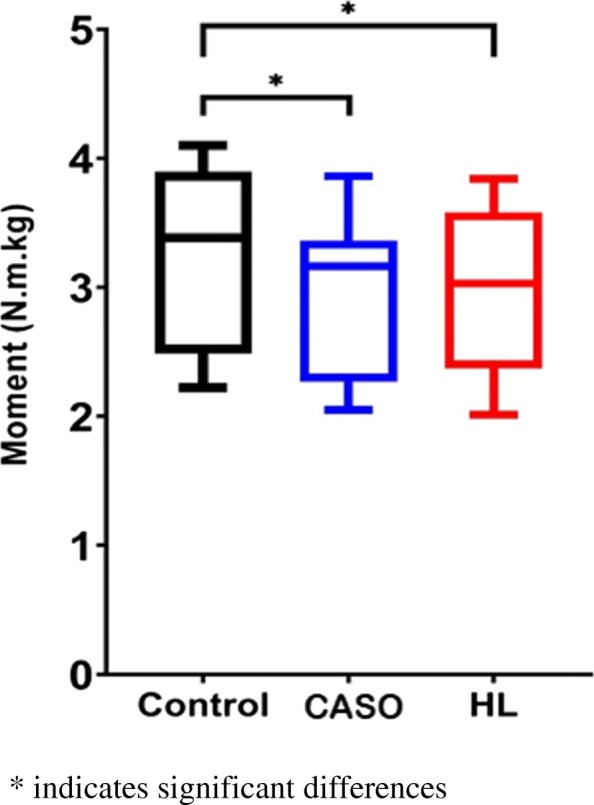
Peak Plantar Flexion moment in the three conditions

There was significant a difference among the three conditions, F (2, 22) = 21.29, *P* < 0.001. Post-hoc comparison showed that there was significant reduction in peak plantarflexion moment when running with CASO (*p* = 0.001, d = 0.44) and HL (p = 0.001, d = 0.39) compared with no orthotic intervention. Although there is no significant difference between the two orthoses, the peak plantarflexion moment from using CASO was slightly lower than using HL in running (*p* > 0.05, d = 0.04) (Fig. 8).

### Peak Achilles tendon load

**Fig. 9:**
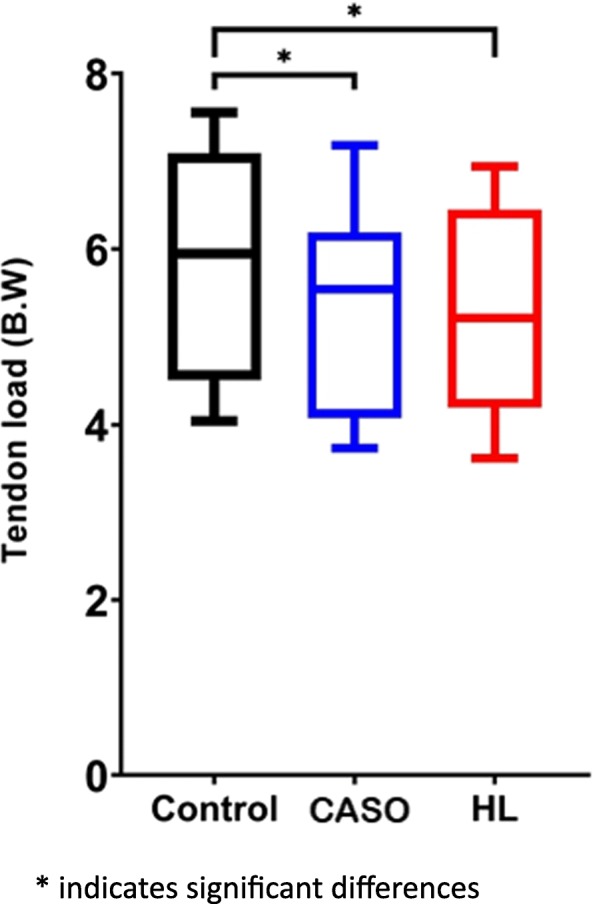
Peak Achilles tendon load in the three conditions

Significant difference was found among the three conditions, F (2, 22) = 25.02, *P* < 0.001. The Post-hoc comparison revealed that the Peak ATL was significantly reduced when using CASO (*p* = 0.001, d = 0.43) and HL (p = 0.001, d = 0.48) during running than the control having no orthotic intervention in the post-hoc test. There was no significant differences (p > 0.05, d = 0.05) between CASO and HL while the Peak Achilles tendon load of using HL was slightly lower than that of CA (Fig. 9).

### Time to peak Achilles tendon force

**Fig. 10:**
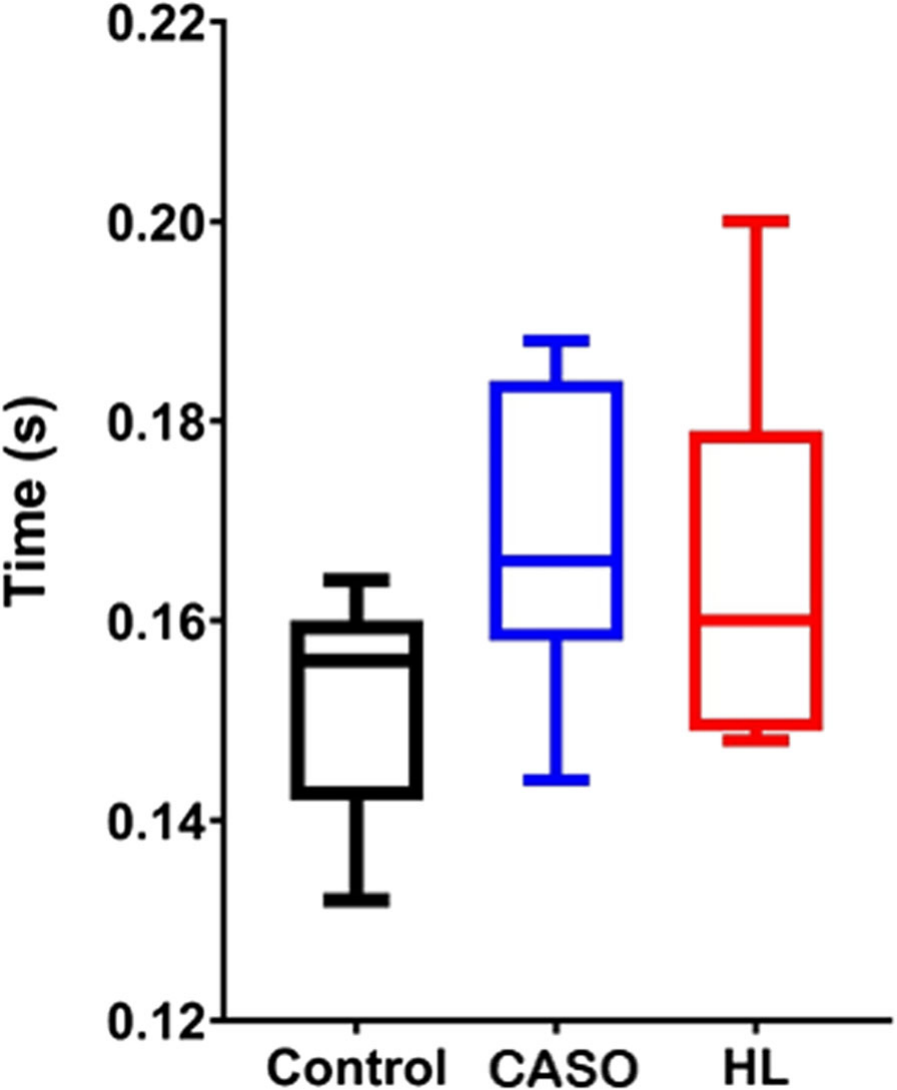
Time to peak Achilles tendon force in the three conditions

Significant difference was found within the three conditions, F (2, 22) =4.90, p = 0.001, in which the difference between CASO (*p* = 0.56, d = 0.13) and the control was not statistically significant. No significant difference (*P* > 0.05) was also shown between the two orthotic interventions (*p* = 0.83, d = 0.28) and between control and HL(*p* = 0.24, d = 0.87) in the post-hoc comparison. When compared with the control, both CASO and HL intervention demonstrated a slightly longer time required from initial contact to peak Achilles tendon force (Fig. 10).

### Achilles tendon loading rate

**Fig. 11:**
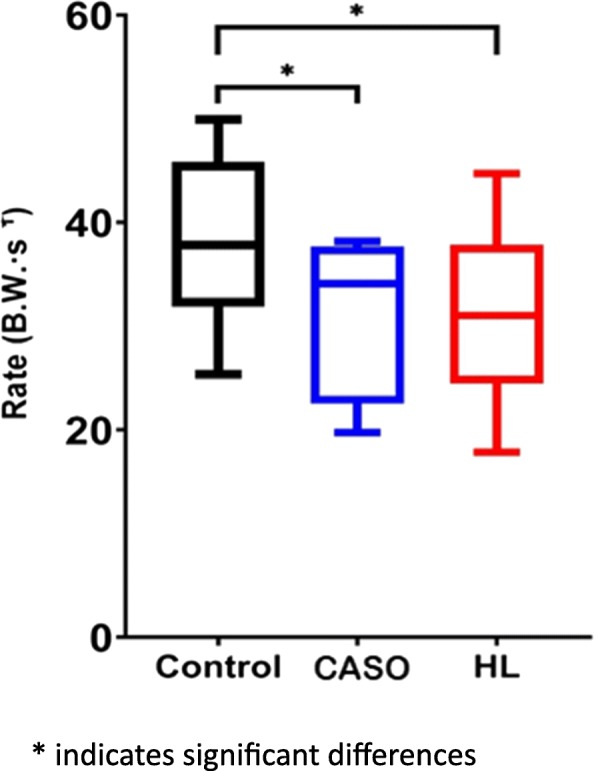
Achilles tendon loading rate in the three conditions

Repeated measures ANOVA indicated a significant difference within the three conditions, F (2, 22) =15.38, *p* < 0.001. Running with CASO (*p* = 0.003, d = 0.93) and HL (*p* = 0.004, d = 0.78) resulted in significantly lower ATLR when compared with the control group (Table 2). While the difference between CASO and HL was not statistically significant (*p* > 0.05, d = 0.09), the use of CASO yielded a slightly lower loading rate than that of HL (Fig. 11).

### Peak dorsiflexion angle

**Fig. 12:**
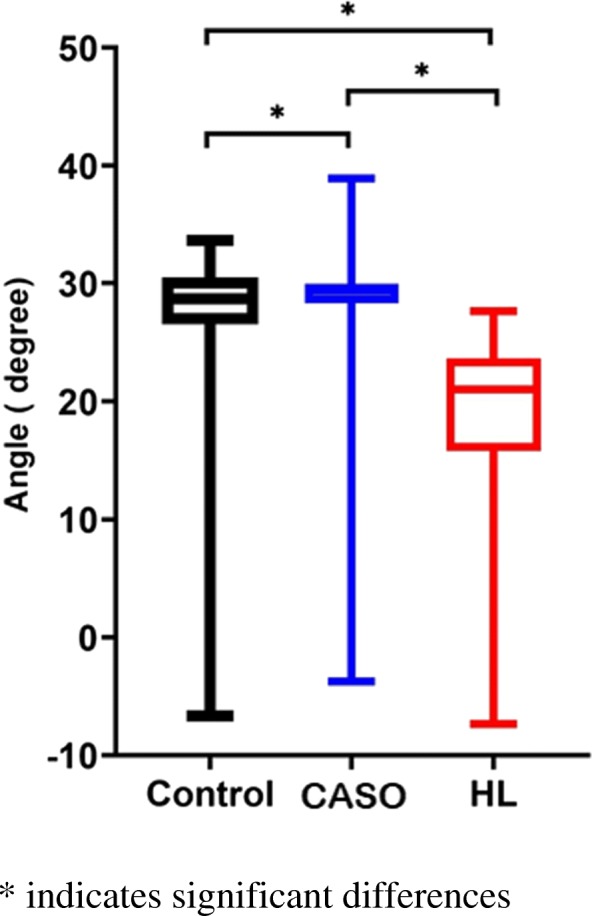
Peak dorsiflexion angle in the three conditions

There was a significant difference found between the three conditions, F (2, 22) = 69.76, *p* < 0.001. Post-hoc comparison also demonstrated significant difference within the three conditions in which CASO and HL resulted in an increase (*p* = 0.042, d = 0.14) and a decrease (*p* < 0.001, d = 0.72) in peak dorsiflexion angle respectively while the peak dorsiflexion angle (*p* < 0.001, d = 0.87) of using CASO was higher than that of HL (Table 2) (Fig. 12).

## Discussion

The current study aimed to investigate the effect of the Customized arch support orthoses (CASO) and orthotic heel lift (HL) on the reduction of ATL for flatfeet runners during running activities. Previous studies mainly focused on the treatment outcomes of orthotic devices towards Achilles tendon pathology, while only a few studies examined the changes in ATL and the results were quite diverse. To the best of our knowledge, this represents the first study to quantify the two currently employed orthotic treatment options, CASO and HL, for Achilles tendinopathy in terms of the effect on ATL.

Our results showed that ankle kinetics in the sagittal plane was reduced by using orthotic interventions. The primary outcomes, peak ATL and peak ATLR, were significantly reduced with the two presence testing orthoses, by 9.1 and 10.3% for CASO and HL respectively when compared to running with shoe only. While the difference between CASO and HL on ATL as well as the loading rate were not statistically significant, HL presented a slightly higher reduction on peak loading when compared to that of CASO. Whereas CASO just showed a relatively lower peak ATLR than that of HL, which was attributed to a longer time from the initial contact to peak Achilles tendon force being recorded.

### CASO

Considering the application of CASO in Achilles tendinopathy management, [15] first explored the effect of semi-rigid, individual fitted arch support orthoses in runners with Achilles tendinopathy, there was significant symptomatic relief after orthotic intervention as reported through the pain scores. Similar to Mayer et al’s study, Donoghue et al. showed improvements in pain symptoms with the use of custom foot arch support orthoses were reported from chronic Achilles tendinopathy runners. Besides, Sinclair et al. first investigated the effect of a commercially available arch support insole on ATL of 12 runners, the result showed a significant reduction in ATL for runners using arch support insole.

In this study, apart from the main reduction in ATL and ATLR, the mean peak plantarflexion moment was reduced by 9.3% and the peak dorsiflexion angle was increased by 6.0%. These were consistent with the result of previous studies [19, 37] where a decrease in peak plantarflexion moment, as well as an increase in peak dorsiflexion angle were found during stance phase when using foot orthoses comparing to the control group. This indicated that CASO had a significant effect on the angle and moment of the ankle joint in the sagittal plane during the stance phase of running. Comparing to a normal foot, [38] found out that people with midfoot pronation exhibit a greater peak plantarflexion ankle moment in the stance phase of the gait cycle because of their abnormal biomechanical changes. In view of this, [39, 40] have already demonstrated in their studies that by using appropriate orthotics design, these features could be corrected. These findings were similar to our current results which indicated CASO had the efficacy to improve the flat foot running gait pattern.

The reduction of ATL parameters from CASO was not only attributed to the decrease in peak plantarflexion moment but also the increase in dorsiflexion angle according to our algorithm model. It is hypothesized that it was caused by the additional midfoot support and midsole cushioning from CASO. Runners would elect to increase dorsiflexion angle at heelstrike and throughout the stance phase owing to the increased in midsole support [41], this, in turn, lengthened the moment arm of the Achilles tendon, thus reducing the loading of Achilles tendon [19]. Moreover, people with the pronation problem would suffer from reduced ankle flexibility because of the tightness in gastrocnemius or soleus, which increase the risk of Achilles tendinopathy. This was due to the prolonged contraction of the gastro-soleus complex in order to control pronation [42]. Accordingly, correcting the midfoot pronation by the use of CASO might help relieve the tightness of the gastroc-soleus complex to improve the flexibility in the ankle, giving rise to the increase in dorsiflexion angle in the present finding. Limited ankle joint dorsiflexion also induced gait changes of shorter midstance period, this indicated increased in joint dorsiflexion angle by CASO in relation to the increased in the timing of peak loading. However, since only the immediate effect of the orthotic intervention was reflected in our investigation, the long-term effect of CASO should be explored to confirm such relation.

### HL

With respect to the effectiveness of HL, Farris et al. evaluated different height of HL on ATL in female runners which showed that an 18 mm heel lift yielded a better outcome in reducing ATL and strain during running than that of using 12 mm one [22]. A similar result was found by Wulf et al. [20], the tensile load in Achilles tendon was examined through ultrasound transmission speed, the ATL was lower with the addition of a 12 mm heel lift in 12 male runners. Rabusin et al. also reported that an orthotic heel lift had the same effectiveness as calf muscle eccentric exercise for Achilles tendinopathy management in a randomized controlled trial [16].

The results of recent findings of the efficacy of HL were relatively similar to those of CASO. Besides reducing the ATL and ATLR, the mean peak plantarflexion moment was reduced by 8.7%, the peak dorsiflexion angle was decreased by 28.2%. When looking at the previous studies about the effect of HL on ATL, the results were quite diverse and varied among individuals. Dixon and Kerwin found an increase in ankle joint moment and Achilles tendon forces when patients wore 7.5 mm and 15 mm heel lifts in comparison to the barefoot condition but the time to peak force and rate of loading were found lower in the follow-up study [21]. It was contrary to our results in which a decrease in mean ankle joint moment and Achilles tendon force resulted. When comparing individually, only 1 out of 12 participants demonstrated a slightly higher peak Achilles tendon force with respect to the control condition. One of the reasons leading to these variations might due to the different thickness and the material used for HL. Given the previous research showing positive results, the magnitude of HL required is greater than the HL used for reducing pain and injury [44]. Thickness of 18 mm HL was used in the current study, according to the study done by Farris and coworkers, 18 mm heel lift significantly reduced both the force and strain in the Achilles tendon during running which reduce the strain in the tendon even better than that of 12 mm heel lift [22]. In addition, Reinschmidt et al. showed that with the increase in heel height, there were 2 out of 5 participants who experienced a decrease in plantarflexion moment during running [43] which was similar to our current findings. This indicated that different heel height had an influence on ankle kinetics. Besides, the difference in density and material of HL between the present and previous studies might affect the shock absorption property, thus affecting the plantarflexion moment and loading rate. Therefore, the large variations of materials and thickness for HL in previous studies might account for the inconsistencies of tendon loading as well as outcomes of the current study.

Although the positive outcomes of using HL in Achilles tendinopathy management are controversial, there was one theory suggesting that heel inserts change the orientation of the foot by raising the heel relative to the forefoot, which helps lower the maximum dorsiflexion angle during the mid-stance phase of gait. It was hypothesized to lessen the eccentric force, which used to control the downward movement of the center of mass to the tendon [10]. Our result showing a decrease in mean maximum dorsiflexion angle when using HL was in line with that theory, suggesting the mechanism of reducing Achilles tendon force.

### CASO and HL

As mentioned above, both CASO and HL demonstrated positive results in the primary outcome, ATL and ATLR, with respect to the control. When comparing the two orthotic interventions, it was not statistically significant for both ATL and rate, 1.3% reduction for HL and 2.2% reduction for CASO respectively. The small differences might be explained by the fact that only sagittal plane kinetic and kinematic were considered due to the current method used in ATL calculation, this was regardless of the fact that the use of CASO and HL would also influence frontal plane parameters like the rearfoot angle and the inversion/eversion moment. Besides, the subtle difference might be owing to the sample size.

The small difference indicated that no relative superiority between these two orthoses could be proved in the present study. In other words, prescriptions of these two orthoses for Achilles tendinopathy management can be considered for individual accommodation so that subjective feedback such as comfort, footwear and sports adaptation can be taken into account for better clinical outcomes. In future studies, in order to further address the differences between CASO and HL, long-term effect and the corresponding muscle activities of triceps surae in related to CASO and HL intervention during running are suggested for investigation.

### Limitations

There are several limitations of this study that warranted being addressed in the future study. First of all, despite our results showing quite a positive outcome of orthotic intervention towards the reduction of ATL, it is only limited to the immediate effects of these two orthoses. It is not clear whether these effects will persist or change over a longer accommodation period. Hence, further study is needed to investigate the long-term effects and the consistency of the efficacy of current orthoses in ATL.

Besides, assumptions are made for the calculation of the Achilles tendon force in our current analysis such that all other structures inducing the internal moment are neglected. Of which, the muscle activity of triceps surae during running have not taken into account. However, triceps surae generates most of the net force during dorsiflexion which applies across the Achilles tendon to control ankle movement. Therefore, the force from triceps surae also has an effect on the ATL [43]. Recent studies also suggested that altered neuromotor control from triceps surae would be one of the underlying mechanisms leading to Achilles tendinopathy [18]. In view of these, further investigation including the EMG signals from triceps surae together with ankle kinetic and kinematic measured would provide more comprehensive information about the effect and difference of CASO and HL on the reduction of tendon loading.

Regarding the current findings, there was not statistically significant as well as a small confidence interval were found between the two orthotic intervention in ATL and ATLR, with the difference in 95% confidence interval extends from − 0.17 to 0.30 respectively. This range includes zero, which indicates that the difference is not statistically significant. The underlying reason behind might due to insufficient sample size used in current study such taht a larger sample size is required in future study.

For the measurement of kinematic data, owing to some of the reflective markers attached to the vamp of the shoe instead of directly onto the skin, it is difficult to ensure the trajectories acquired from the externally- mounted reflective markers coincide with those of the internal foot structure accurately and consistently, thus certain of errors might have resulted.

### Clinical relevance

Flatfeet is recognized as a contributing factor in various lower limb musculoskeletal pathologies in running sports including Achilles tendinopathy. Foot orthoses such as CASO and HL have been commonly used for the management of Achilles tendinopathy which yield a positive clinical outcome. Although the underlying reasons for the efficacy of foot orthoses on Achilles tendinopathy are still unknown, load reduction on the tendon is one of the proposed mechanisms. By quantifying the effectiveness of CASO and HL in terms of ATL in the present study, positive results were found when compared to the control. This provides better insight and evidence of the efficacy for commonly used orthotic intervention in Achilles tendinopathy management. In other words, the use of CASO and HL in running activities may be considered as a preventive measure for flatfoot runners who are at a higher risk of Achilles tendinopathy. At the same time, the findings may also act as a practice guideline for orthotic prescriptions in clinical settings.

## Conclusions

The findings from the current study showed that both CASO and HL were able to significantly reduce the ATL and ATFR for runners with flatfeet in running. While there are subtle differences in the Achilles tendon kinetic parameters, no relative superiority of between the two types of orthoses could be concluded. Owing to the proposed correlation between ATL and Achilles tendinopathy, we speculated that the use of CASO and HL may attenuate the risk of developing running related pathologies in AT.

## Data Availability

The datasets used and/or analysed during the current study are available from the corresponding author on reasonable request.

## Abbreviations

ATL: Achilles tendon load
ATLR: Achilles tendon loading rate
CASO: Customized arch support orthoses
FPI: Foot posture index
HL: Orthotic heel lift
